# Dynamic Tracking of State Anxiety *via* Multi-Modal Data and Machine Learning

**DOI:** 10.3389/fpsyt.2022.757961

**Published:** 2022-03-02

**Authors:** Yue Ding, Jingjing Liu, Xiaochen Zhang, Zhi Yang

**Affiliations:** ^1^Laboratory of Psychological Health and Imaging, Shanghai Mental Health Center, Shanghai Jiao Tong University School of Medicine, Shanghai, China; ^2^Institute of Psychological and Behavioral Sciences, Shanghai Jiao Tong University, Shanghai, China; ^3^Brain Science and Technology Research Center, Shanghai Jiao Tong University, Shanghai, China

**Keywords:** state anxiety, machine learning, quantitative modeling, dynamic tracking, physiological feature, psychological feature

## Abstract

Anxiety induction is widely used in the investigations of the mechanism and treatment of state anxiety. State anxiety is accompanied by immediate psychological and physiological responses. However, the existing state anxiety measurement, such as the commonly used state anxiety subscale of the State-Trait Anxiety Inventory, mainly relies on questionnaires with low temporal resolution. This study aims to develop a tracking model of state anxiety with high temporal resolution. To capture the dynamic changes of state anxiety levels, we induced the participants' state anxiety through exposure to aversive pictures or the risk of electric shocks and simultaneously recorded multi-modal data, including dimensional emotion ratings, electrocardiogram, and galvanic skin response. Using the paired self-reported state anxiety levels and multi-modal measures, we trained and validated machine learning models to predict state anxiety based on psychological and physiological features extracted from the multi-modal data. The prediction model achieved a high correlation between the predicted and self-reported state anxiety levels. This quantitative model provides fine-grained and sensitive measures of state anxiety levels for future affective brain-computer interaction and anxiety modulation studies.

## Introduction

Anxiety is a mental state of elevated apprehension, arousal, and vigilance usually elicited by the anticipation of threat (1, 2). The defensive response caused by anxiety enables the organism to avoid or reduce harm to ensure its survival (3). But excessive or inappropriate anxiety can become an illness and diminish life quality (4). Anxiety disorder ranks among the most prevalent mental illnesses overall the world (5). A better understanding of the neural mechanism and biomarker of anxiety may benefit the large anxious population (6).

Meta-analysis studies have suggested overlapping neurobiological mechanisms across induced and pathological anxiety (7), allowing for investigations of anxiety by inducing state anxiety (8). Through anxiety induction procedures, one can reproduce concrete transitory anxious states under controlled conditions (9) by using particular situations or stimuli (10–12). Paradigms, such as exposure to disturbing pictures (13, 14) or anticipatory threats [e.g., electric shock; (15)], and experimental situations of failure (12) have been commonly used to induce anxiety. Regardless of the paradigms, a convenient, timely, and objective marker to indicate state anxiety changes is essential.

There have been a series of self-report measures of anxiety that are well-accepted in both clinical and research settings, including the State-Trait Anxiety Inventory (STAI), the Beck Anxiety Inventory (BAI), and the anxiety subscales of the Hospital Anxiety and Depression Scale, etc. (16). Among them, only the state anxiety subscale of STAI (STAI-S), evaluates the current state of anxiety by asking how respondents feel “right now”. STAI-S applies 20 items to measure respondents' subjective feelings of apprehension, tension, nervousness, worry, and activation/arousal of the autonomic nervous system. However, the measuring interval of STAI ranges from 1 h to 104 days (16), lacking the capability of measuring transitory states in a finer temporal resolution. Immediate Mood Scaler (IMS), a newly proposed self-report tool, could capture current mood states with 22 items but is designed for a daily report with a maximum usage frequency being twice a day (17). Though state anxiety is associated with transient sympathetic activation and vagal deactivation (18), most anxiety-related indicators were reported in discrete time points with long intervals (19, 20). There's a lack of a dynamic and transient indicator of state anxiety.

Anxiety is an emotion (21). Emotional states can be described by valence, arousal, and dominance (VAD), ranging from unpleasant to pleasant, from calm to agitated, and from submissive to dominant, respectively (22). In the classic emotional theoretical model, anxiety features high arousal, low valence, and low dominance (23, 24). But to our knowledge, the quantitative relationship between VAD and state anxiety remains unclear.

Apart from self-report tools, physiological responses accompanying anxiety include sweating, heart palpitations, faster and shallower breathing, etc. (25, 26). Studies have revealed several qualitative cardiovascular, electrodermal, and respiratory features associated with anxiety (27), such as increased heart rate, decreased heart rate variability (28, 29), increased skin conductance response and increased skin conductance level (28, 30), increased respiratory rate, as well as decreased tidal volume (30, 31). By combining these physiological features, researchers have been able to differentiate anxiety from other emotional states, such as relaxation, excitement, and fun (32), or identify discrete anxious levels (33). However, the quantitative representations of state anxiety based on the psychological and physiological response are still lacking, limiting the dynamic tracking of the continuously changing anxious levels in anxiety induction paradigms.

The current study aimed to bridge the gap between psychological and physiological response and state anxiety by building regression models of state anxiety based on the combination of dimensional emotion scales and physiological data. We measured the STAI-S among healthy participants discretely before and after emotion induction tasks while recording VAD, electrocardiogram (ECG), and galvanic skin response (GSR) dynamically throughout the whole experiment. We examined whether state anxiety levels can be quantitatively and dynamically evaluated using multi-modal measures.

## Methods

### Participants

Thirty graduate students (15 females, mean age = 24.37 ± 2.16 years) participated in the study. All participants were recruited from colleges and universities, and with normal hearing, normal or corrected-to-normal vision, while without any history of mental disorders or severe physical illnesses. Before the formal experiment, we also collected participants' BAI (mean score = 26.07 ± 5.34, ranging from 21 to 39), a common clinical scale for the diagnosis of anxiety disorder, to ensure that no subclinical patient was included. The study was conducted in accordance with the Declaration of Helsinki and approved by the local ethics committee (SMHC-IRB: 2018-46). Participants gave their written informed consent and received monetary compensation for their participation. Two participants were excluded from the analysis because of lost behavioral data. Another three participants were excluded from the regression analysis because of incomplete physiological data due to technical issues.

### Experimental Procedure and Data Recording

**Figure 1:**
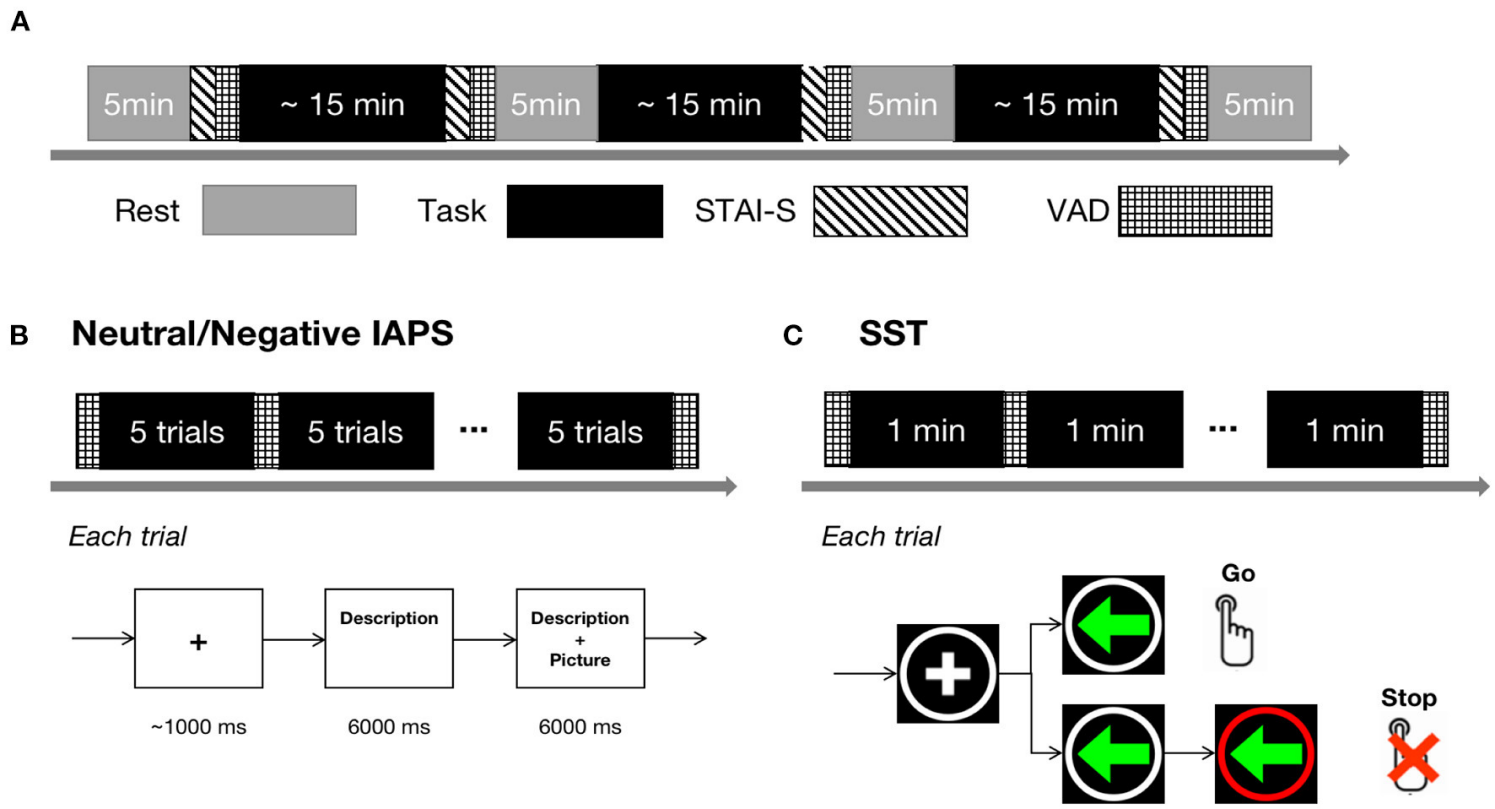
Experimental procedure. **(A)** Overall experimental procedure. The gray blocks represent rest conditions and the black blocks represent task conditions, including Neutral IAPS, Negative IAPS, and SST. The blocks indicated with diagonal lines represent STAI-S sessions and the blocks marked with grids represent VAD rating sessions. **(B)** Experiment design of Neutral/Negative IAPS. The upper panel shows the temporal structure of a block and the bottom panel shows the temporal structure of a trial. **(C)** Experiment design of the SST task. The upper panel shows the temporal structure of a block and the bottom panel shows the temporal structure of a go or stop trial.

An overview of the experimental procedure is shown in Figure 1A. The participants completed three tasks, including two anxious-mood-induction conditions and one control condition. The three tasks were arranged in pseudo-randomized orders across participants. One possible order, in which the control condition located in the middle of the two anxious-mood-induction conditions, was avoided to ensure a fair comparison of the two anxious-mood-induction conditions. The task blocks were padded with 5-min resting blocks.

In the control condition, 65 neutral pictures with medium arousal and valence from the International Affective Picture System [IAPS; (34)] were used as the stimuli. The mean valence, arousal, and dominance ratings of the neutral pictures were 5.53, 4.69, and 5.67, respectively. Each picture was paired with a brief text in simplified Chinese describing the content of the picture (e.g., a picture of people around a market was described as: “People make purchases in the crowded market”). In each trial, the description of a particular picture was presented to participants first and lasted for 6 s, and then the picture appeared while the description remained on the screen for another 6 s (Figure 1B). After every five trials, participants' instantaneous mood in terms of VAD was recorded. The task was referred to as “Neutral IAPS” in the following text.

One of the anxious-mood-induction tasks adopted the same paradigm as the Neutral IAPS but used 65 negative pictures with high arousal and low valence from the IAPS as the stimuli. The mean valence, arousal, and dominance ratings of the negative pictures were 1.99, 6.06, and 3.38, respectively. The brief text description emphasized the lack of control over the anxious scene presented in the pictures [e.g., a picture of a dying person in the bed with a weeper beside was described as: “No one is immune from illness or death, and worse, there may be no one to accompany you.”; (14)]. The task was referred to as “Negative IAPS” in the following text.

The other anxious-mood-induction task was the stop-signal task (35, 36) with electric shock punishment. Two kinds of trials, go trial and stop trial, were randomized evenly in the total 90 trials. As shown in Figure 1C, each trial began with a fixation cross in a white circle presented on a black background in the center of the screen with a duration of 200–400 ms. In the go trials, a left/right green arrow then appeared in the circle. Participants were instructed to respond as quickly and accurately as possible within 500 ms according to the left/right arrows by pressing the left/right buttons (Instruction: “You're going to see an arrow in each trial pointing to left or right. When it occurs, press left or right button correspondingly as soon as possible.”). In the stop trial, the white circle turned red within a variable delay of 50–450 ms after the left/right green arrow appeared. The participants were told that when seeing the white circle turning red, they should not press the button (Instruction: “When the white circle around the arrow turns to red, you're not allowed to press any button.”). Wrong responses (too slow, failed to stop, or wrong key) would cause a warning text in the circle as well as an electric shock. During the task, after every minute, the VAD ratings were recorded. The task was referred to as “SST” in the following text. Before starting the SST, the participants must complete 20 consecutive practice trials, where they solely needed to press the left/right buttons as quickly and accurately as possible within 500 ms according to the left/right arrows without stop signs or electric shock punishment.

The intensity of the electric shocks was individually determined before the experiment. The participants rated their uncomfortable feelings from 1 to 9 (a higher score means more unbearable feeling) when they received electric stimulation. The current intensity started from 300 μA and increased in steps of 200 μA until the participant's rating was equal to or larger than 8 or the current intensity reached 5 mA. Then the personalized intensity range was divided evenly into 15 current intensity levels and presented to the participants in randomized orders. The participants again assessed how they felt under these levels of stimulation. After fitting the ratings to the current intensity linearly, the intensity level corresponding to each participant's rating of 7 points was used as the individual's stimulation intensity (intensity = 2,516 ± 1,269 μA).

In the resting blocks, the participants were asked to look at the fixation and rest for 5 min. The first resting block was at the beginning of the experiment, followed by a STAI-S and VAD rating. In the other three resting blocks, the STAI-S and VAD ratings were conducted before the rest period.

### Assessment Tools

The experiment was carried out in a laboratory environment. The stimuli were displayed on an LCD monitor (15.6-inch) with a 60 Hz refreshing rate, placed at a distance of around 60 cm from participants. Presentation of the stimuli and the rating procedure were programmed in MATLAB (The Mathworks, USA) using the Psychophysics Toolbox 3.0 extensions (37).

The psychological data, including STAI and VAD, was recorded by custom MATLAB programs. STAI is a commonly used measure of anxiety via self-reporting the presence and severity of current symptoms of anxiety (STAI-S) or a generalized propensity to be anxious (STAI-T). Test-retest reliability coefficients on the initial development of STAI ranged from 0.31 to 0.86, and the internal consistency alpha coefficients ranged from 0.86 to 0.95 (38). The validity of STAI-S was initially derived from testing in situations characterized by high state stress (16). Aligned with classic emotional studies (23, 24), the variance in emotional assessments was accounted for by three major dimensions: affective valence, arousal, and dominance (39), by a series of the Self-Assessment Manikins (SAM) scales using 9-point scales (1–9).

The physiological data, including ECG and GSR, was recorded throughout the whole experiment with the NeuSen W system (Neuracle, China) at a sampling frequency of 1,000 Hz. To measure ECG, self-adhesive and disposable ECG electrodes with conductive gel attached were placed onto the inside of wrists on both sides. The analog ECG signals recorded by the operational amplifier (OPA precision operational amplifier, TI.) were converted into digital signals through a 16-bit A/D converter (ADS analog-to-digital converter, TI.). The acquisition of GSR was obtained by applying a constant voltage (≤3.3 V) to record the change of skin impedance (40) through two Ag/AgCl electrodes pasted on the middle phalanges of the index and middle fingers of the non-dominant hand, to get the digital skin electrical signals. The digital skin electrical signals were then converted from voltage value to micro Siemens by GSR recorder 2.0 (Neuracle, China). The measurement range is 10 kΩ-5.0 MΩ (0.2–100 μS), while the frequency range is DC to 15.9 Hz.

### Data Preprocessing and Feature Extraction

Data were segmented into epochs before preprocessing. For the rest condition, the data of each 5-min block was taken as an epoch. For the Neutral and Negative IAPS tasks, the data of every five pictures was regarded as an epoch, which was about 1 min long. For the SST task, the data between every two VAD ratings was considered an epoch, lasting about 1 min. The multi-modal data was analyzed epoch-by-epoch using custom MATLAB scripts. The preprocessing and feature extraction of ECG was conducted using HRVTool, a MATLAB toolbox for analyzing heart rate variability [HRV; (41)], while GSR data was analyzed using another MATLAB toolbox—Ledalab (42).

**Figure 2:**
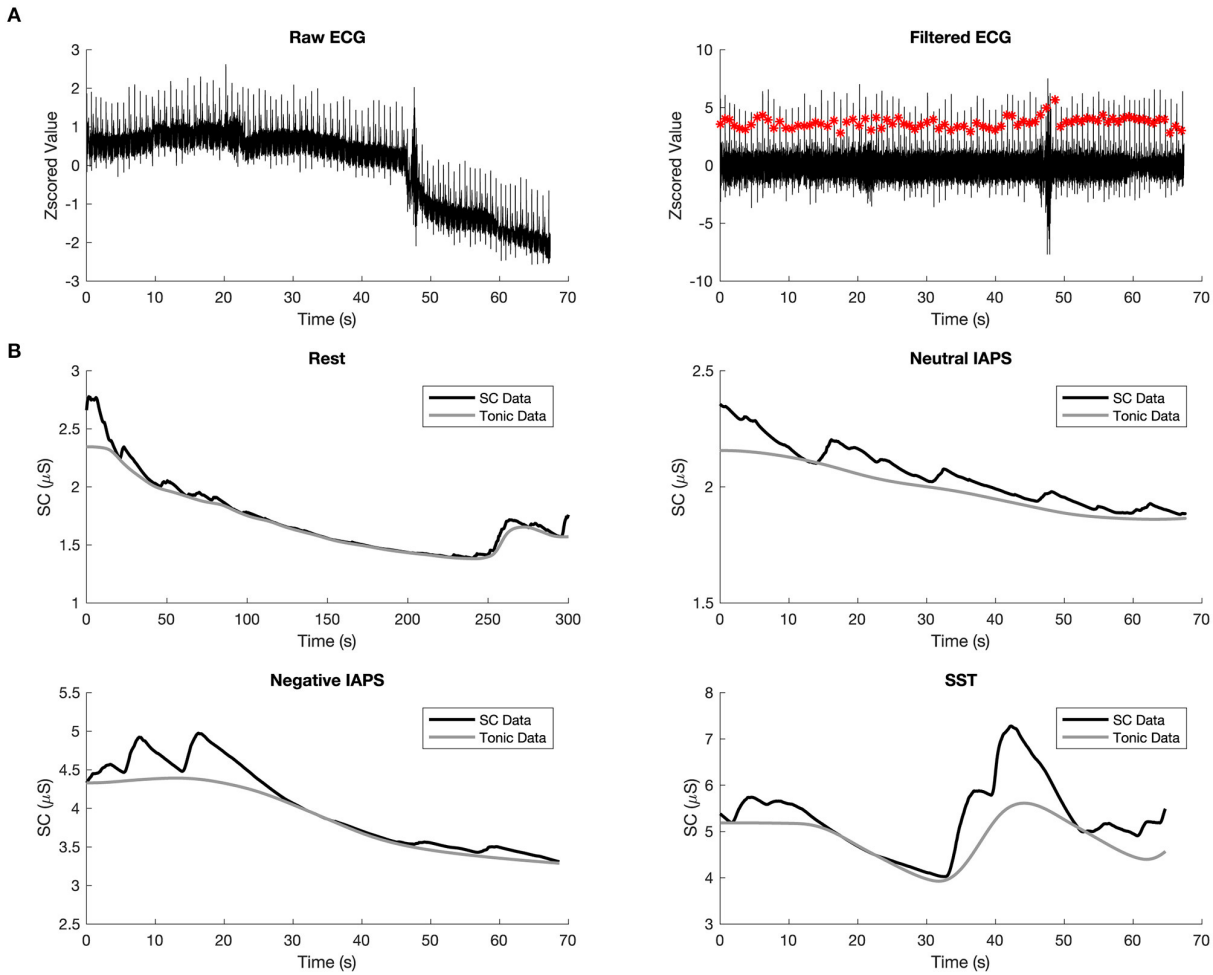
Preprocessing of ECG and GSR. **(A)** Example waveform of raw ECG (left) and filtered ECG (right), the red dots indicate detected beats. **(B)** Example waveform of denoised skin conductance data (in black) and the tonic component of GSR (in gray) in rest (top left), Neutral IAPS (top right), Negative IAPS (bottom left), and SST (bottom right).

The raw ECG signals were filtered by a trimmed moving average filter with the window length of 0.2 s and the trimming percentage of 0.25 to remove the muscle artifacts that generally come from hands or arms movements. The filtered signals were Z-scored before computing the beat annotations. The heartbeats between 50 and 220 beats per minute were taken as valid beats, and the R-R intervals of QRS waves were calculated based on these signals (Figure 2A). Relative R-R intervals were calculated to remove artifacts from the R-R sequences. After removing the artifacts, fifteen HRV features were extracted, including: (1) the mean and (2) the standard deviation of R-R intervals; (3) the median and (4) the interquartile range (annular intensity) of the Euclidean distance to the center point of the return map of relative R-R intervals (43); (5) the root mean square of successive differences of R-R intervals (RMSSD); (6) the probability of the successive R-R differences exceeding 50 ms; (7) the reciprocal of the probability of the highest bin of the histogram of R-R intervals with bin size 1/128, known as the triangular index (TRI); (8) the width of the triangular function, which has the best fit to the sample histogram, known as the TINN value; (9) short-term HRV (SD1) and (10) long-term HRV (SD2), in terms of standard deviations along the identity line and its perpendicular axis of the return map of R-R intervals, also known as Poincare map (44), and (11) SD1-SD2 ratio; Spectral density function of interpolated R-R tachogram, which is basically divided into two bands, low frequency band (LF) and high frequency band (HF): (12) LF (0.04–0.15 Hz) has been contributed from vagal and sympathetic modulation of R-R intervals, (13) HF in HRV (0.15–0.40 Hz) represents a pure vagal efferent signal that is modulated by respiratory sinus arrhythmia, (14) the LF/HF ratio gives an index of autonomic balance, whose high values indicate sympathetic nervous system predominance and low values indicate parasympathetic nervous system predominance; and (15) approximate entropy.

The raw GSR signals were filtered by a Butterworth lowpass filter with a cutoff frequency of 5 Hz and a filter order of 10 (40). The filtered signals were smoothed using the adaptive Gaussian window with a window width no larger than 3 s. The tonic component of one trial was then estimated by Continuous Decomposition Analysis (42). The average mean of the tonic component was taken as the skin conductance level (SCL) and used as the feature of GSR. The four subplots of Figure 2B show the overall skin conductance (SC) data in black and the extracted SCL in gray for the four conditions, respectively. Together with the three-dimensional features of VAD, 19 different features in total were used in the current study.

### Regression Models and Prediction Evaluation

The four rest epochs of 25 valid participants were used to build the prediction model of state anxiety, where STAI-S scores were regarded as the ground truth of state anxiety. Both the extracted features and STAI-S of the first epoch were regarded as the baseline of each participant. The features and STAI-S of the remaining three trials were normalized to the baseline and then Z-scored within each participant for each feature. The latter three resting trials of the 25 participants were used in the correlation analysis and regression, ending with 75 observations in total.

A small portion (<2%) of data failed to reflect valid features for two reasons: SCL being out of the measuring range or too few beats being recognized from ECG. Trimmed scores regression was applied for the missing data imputation via Missing Data Imputation Toolbox in MATLAB (45), with the number of principal components being 3, maximum iteration being 5,000, and tolerance being 10e-10.

The data fed into the regression models contained 75 observations with 19 features, and each observation had a Z-scored STAI-S as the label. Four different regression approaches were explored, including (1) linear regression, (2) support vector regression (SVR), (3) LASSO regression, and (4) ensemble of trees. The regression performance was validated by implementing a Leave One Subject Out (LOSO) scheme. Metrics, including mean absolute error (MAE), root mean squared error (RMSE), coefficient of determination (R^2^), and adjusted R^2^, were recruited to evaluate the performance of the regression models.

## Results

### Behavioral Results

**Figure 3:**
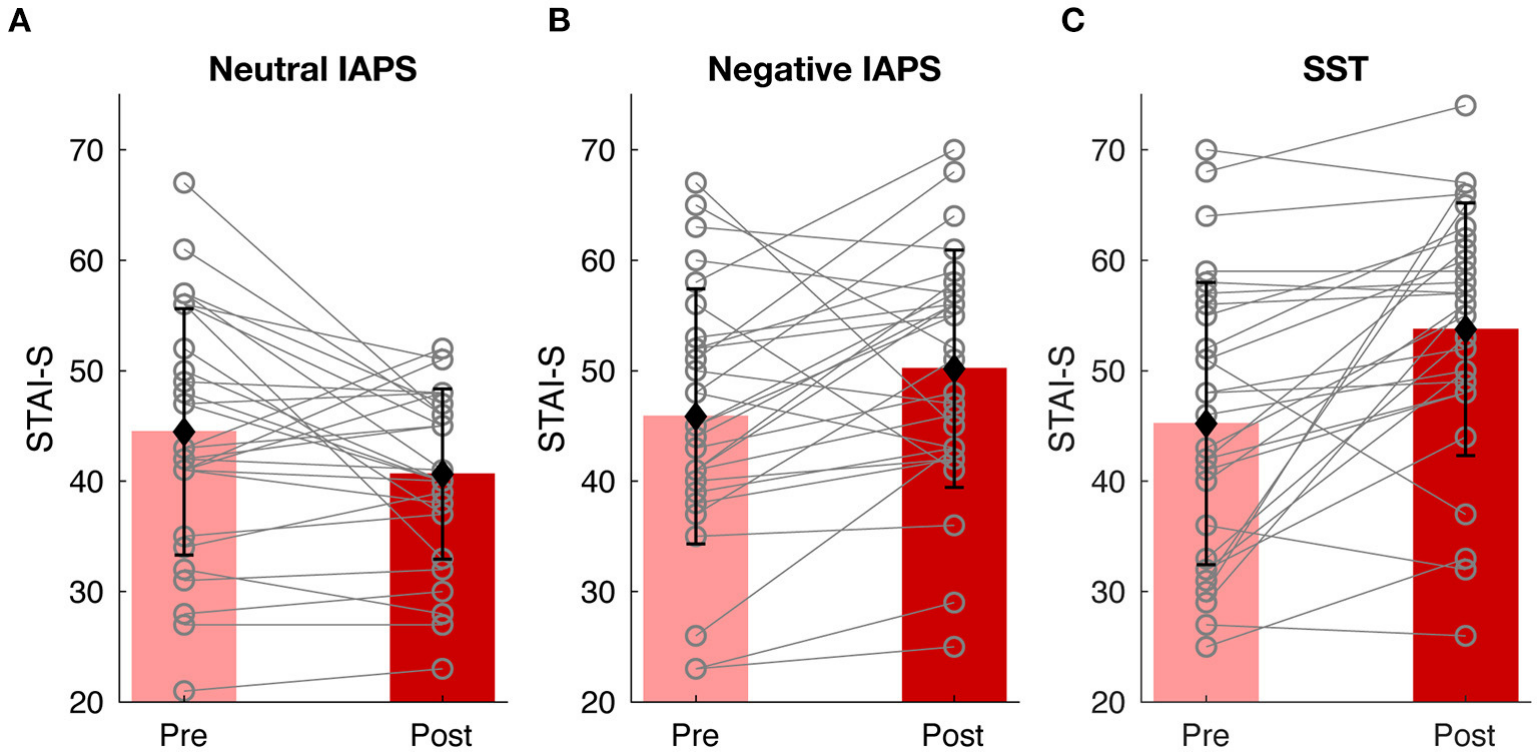
Behavioral results. State anxiety level changes of Neutral IAPS **(A)**, Negative IAPS **(B)**, and SST **(C)**. In each condition, the pink and red bars show the mean value of STAI-S scores before and after tasks, the black error bars represent the standard deviation of STAI-S scores, and the gray lines indicate alteration from pre-task to post-task for each participant.

To ensure the experiment successfully induced state anxiety, the STAI-S scores, as the ground truth of transient anxiety level in the current study, were examined. A one-tail paired *t*-test was conducted to check whether the STAI-S scores increased after tasks compared to before. In Neutral IAPS (Figure 3A), there was no significant difference of STAI-S scores after (M = 40.6429, SD = 7.7038) watching pictures compared to before (M = 44.4643, SD = 11.1471); *t*_(27)_ = 2.3286, Bonferroni adjusted *p* = 1. In contrast, in Negative IAPS (Figure 3B), the STAI-S scores were significantly larger after (M = 50.1786, SD = 10.7464) watching pictures compared to before (M = 45.8571, SD = 11.5461); *t*_(27)_ = −2.2986, Bonferroni adjusted *p* = 0.0442. SST also successfully induced state anxiety (Figure 3C), as reflected by higher STAI-S scores after the task (M = 53.7500, SD = 11.4362), compared to before (M = 45.2143, SD = 12.7812); *t*_(27)_ = −4.1105, Bonferroni adjusted *p* = 0.0008. Among the 28 valid participants, 11 in Neutral IAPS, 21 in Negative IAPS, and 22 in SST showed increased STAI-S scores after the tasks. At the group level, both the anxious-induction tasks successfully induced state anxiety. Even for the participants with non-significant changes, the diverse STAI-S scores throughout the experiment could still be used to explore the relationship between state anxiety level and physiological and psychological features.

### The Correlation Between STAI-S and Physiological and Psychological Features

**Figure 4:**
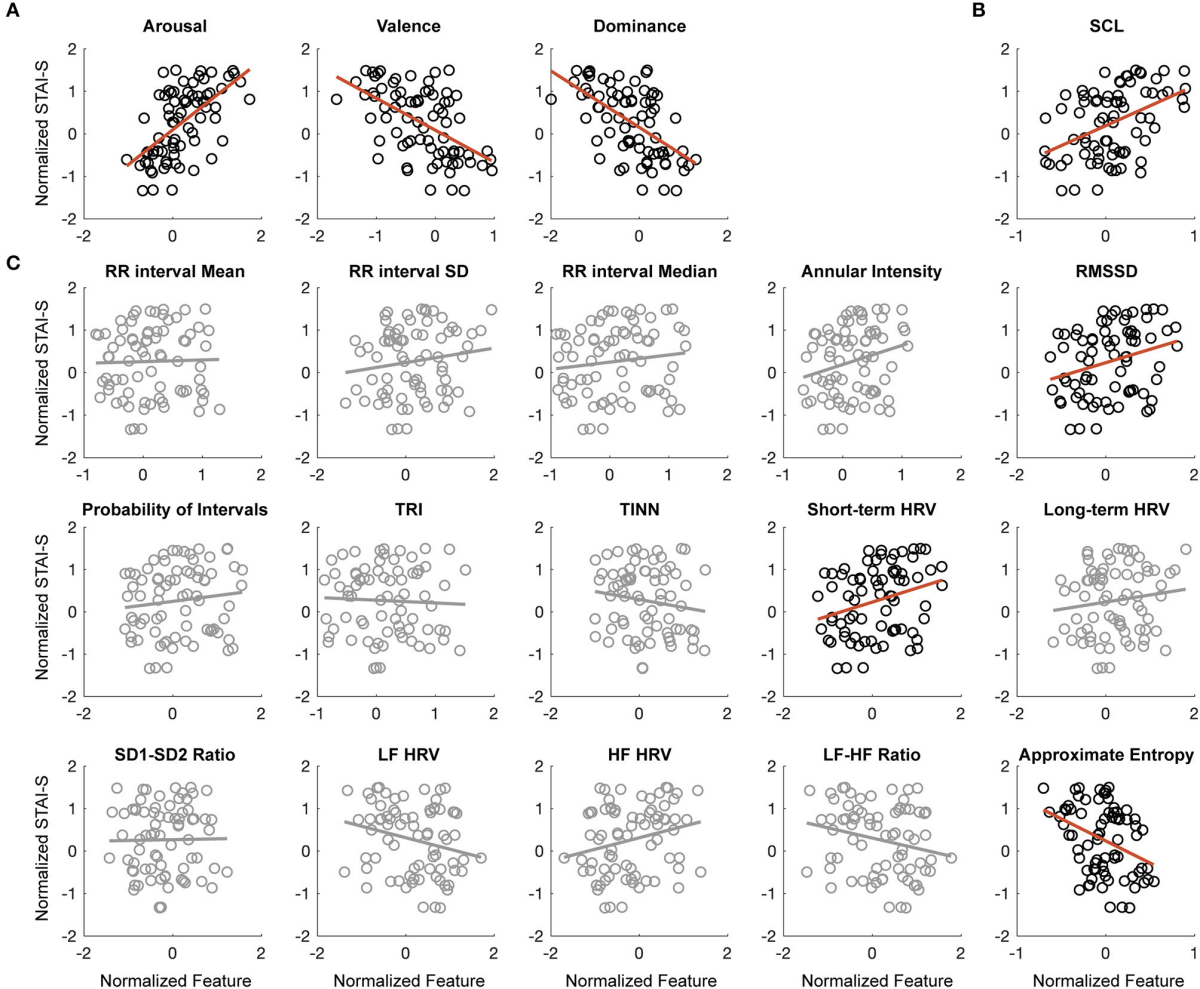
The correlation between STAI-S and physiological and psychological features. **(A)** Correlation between arousal (left), valence (middle), dominance (right) ratings, and STAI-S scores. **(B)** Correlation between SCL and STAI-S scores. **(C)** Correlation between 15 HRV features and STAI-S scores. The circle points are samples and the red lines are the linear fitting lines. The non-significant correlations were shadowed in gray.

To have a global view of the relationships between state anxiety level and physiological and psychological features, Pearson's correlation was conducted to test the linear relationship between individual features and the STAI-S scores. As shown in Figure 4, the three-dimensional features of VAD significantly correlated with SAI scores. Consistent with the classic emotion theory, arousal scores were positively correlated with STAI-S [*r*_(73)_ = 0.5936, FDR-adjusted *p* < 0.001], while valence [*r*_(73)_ = −0.5417, FDR-adjusted *p* < 0.001] and dominance [*r*_(73)_ = −0.5751, FDR-adjusted *p* < 0.001] were negatively correlated. SCL also significantly increased with the increasing of STAI-S [*r*_(73)_ = 0.4418, FDR-adjusted *p* < 0.001]. Among the 15 HRV features, three of them were significantly correlated with STAI-S, including approximate entropy [*r*_(73)_ = −0.3571, FDR-adjusted *p* = 0.0063], short-term HRV [*r*_(73)_ = 0.2868, FDR-adjusted *p* = 0.0399], and RMSSD [*r*_(82)_ = 0.2790, FDR-adjusted *p* = 0.0417]. Though the rest features didn't show significant correlation coefficients (*p* > 0.05), they still might help with the prediction of STAI-S, therefore all the features would feed into the following regression models.

### Prediction of STAI-S Using Multi-Modal Data

Four different regression models were used to predict STAI-S. The prediction performance using all the 19 features, the VAD only or the physiological features only, was compared. Among the four models, LASSO regression achieved superior prediction performance regardless of feature types. When using all the features, the performance of LASSO regression was the best in terms of adjusted R^2^ (Supplementary Figure 1) as well as the other three metrics (Supplementary Table 1).

**Figure 5:**
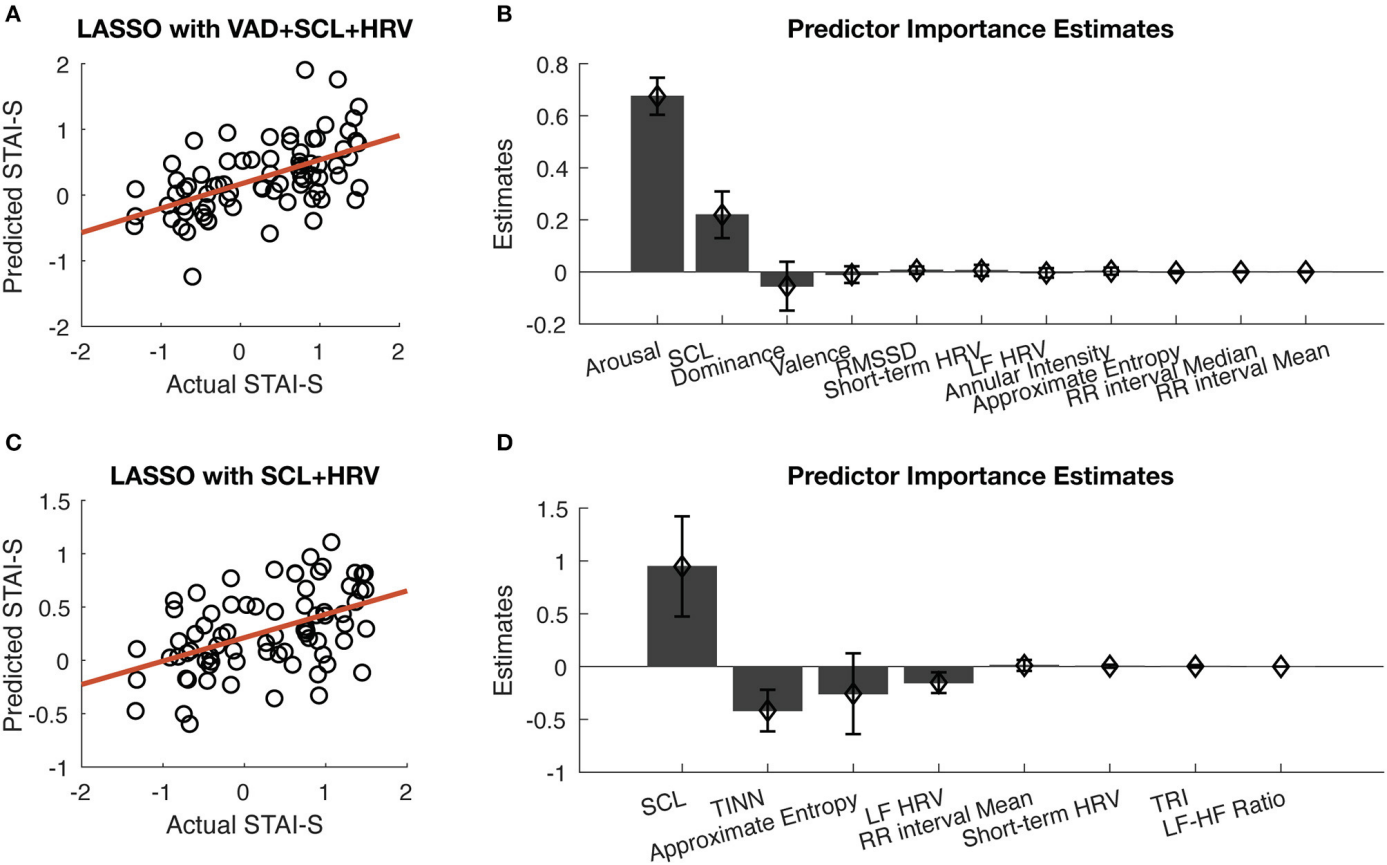
Prediction of STAI-S using multi-modal data. **(A)** Correlation between predicted STAI-S and actual STAI-S using all the features in the LASSO regression model. **(B)** Sorted predictor importance estimates of the LASSO regression model when using all the features. **(C)** Correlation between predicted STAI-S and actual STAI-S using only physiological features. **(D)** Sorted predictor importance estimates of the LASSO regression model when using only physiological features. The error bars represent standard deviation across participants in the “leave one subject out” validation.

As shown in Figure 5A, when using all the 19 features, the correlation coefficient between the predicted STAI-S and actual STAI-S was 0.5528 (Bonferroni adjusted *p* < 0.0001). Among all the 19 features, arousal ratings in VAD contributed most to the prediction in terms of the absolute value of Beta, followed by SCL. The other two dimensions of VAD also contributed more than the features of HRV. Among the 15 features of HRV, eight features, including RMSDD, short-term HRV, LF HRV, annual intensity, approximate entropy, the median and mean of RR intervals, helped with the prediction, but with minor contribution (Figure 5B). When using VAD only, the correlation coefficient between predicted STAI-S and actual STAI-S was slightly worse than using all features (*r* = 0.5523, Bonferroni adjusted *p* < 0.0001).

When excluded VAD, the correlation coefficient between predicted STAI-S and actual STAI-S was 0.4748 (Bonferroni adjusted *p* < 0.0001), as shown in Figure 5C. Among the physiological features, eight features were contributing to the LASSO regression, including major contribution of SCL, TINN approximate entropy, and LF HRV, as well as minor contribution of the mean of RR intervals, short-term HRV, TRI, and LF-HF ratio (Figure 5D). That is, STAI-S could be predicted by HRV and SCL with or without VAD.

## Discussion

The present study aims to build a dynamic tracking model of state anxiety. By eliciting diverse state anxiety levels via classical anxious-mood-induction tasks, multi-modal data, including ECG, GSR, and VAD ratings, was fed into a regression model to predict the state anxiety level with high temporal resolution. By applying machine learning approaches, state anxiety levels can be predicted by combining these multi-modal data. This study not only demonstrates the statistical evidence of the psychological and physiological characteristics of the state anxiety but also proposes a state anxiety prediction model with higher temporal resolution than the traditional measurement, thus providing a practical solution for tracking the dynamic changes of the state anxiety level.

In line with the classical emotion theory (23, 24), the current study confirms that the state anxiety evoked by the anxiety induction tasks has higher arousal, lower valence, and lower dominance. This can be supported by the positive or negative correlation coefficients of these VAD features in the correlation analysis (Figure 4) and the signs of their regression coefficients in the regression model (Figure 5B). Similarly, the findings that SCL, RMSSD, and short-term HRV positively correlated with state anxiety levels and that the approximate entropy of HRV negatively correlated with state anxiety levels echo the existing knowledge (26, 46, 47). The replication of established relationships supports the validity of the current study. Methodologically, the data were divided into a training set and test set, and the prediction capability was validated by “leave one subject out” to ensure the generalization of the prediction model. Although Z-score had been applied to multiple sampling points within participants to diminish the individual difference, “leave one subject out” instead of “leave one sample out” was used in the current study to avoid data leakage by ensuring that the samples from the same participant would be either in the training set or test set. These approaches help to enhance the reliability and generalizability of the findings.

Unlike trait anxiety, state anxiety represents the transient psychological and physiological response to potential threats, and a dynamic tracking system would provide valuable information for anxiety-related studies. The evident convergence between subjective psychological and objective physiological measures, referred to as emotional coherence, has been confirmed in anxiety-related paradigms (26, 48). In our anxiety induction paradigms, anxious emotion coherence across physiological and psychological responses also appeared. Such coordination across physiological and psychological responses relies on the central and peripheral nervous system to a great extent, where the vegetative nervous system accounts for the majority of anxious bodily symptoms. The human body can react to adverse events with a temporary increased sympathetic nervous system to protect itself from harm, exhibiting symptoms like palpitation, sweating, and trembling (49). The interplay between the two subsystems of the autonomic system, namely the sympathetic (SNS) and parasympathetic nerve systems (PNS), cooperates on the stress response to threatening events and forms a central component of state anxiety. Notably, the interplay occurs rapidly to allow the mental states to adapt to the constantly changing environment. Therefore, a time-resolved measure of state anxiety is essential for investigating the anxiety dynamics responding to various environmental factors.

Considering the overlapping neurobiological mechanisms between induced and pathological anxiety (7), inducing state anxiety has been widely used to investigate anxiety (8). Previous studies have provided theoretical evidence that physiological measurement could reflect the changes from before to after the induction (14, 50). That's a statistical foundation for constructing the quantitative prediction model as proposed in this study. In addition to distinguishable physiological characteristics, the prediction model could provide a more quantitative mapping between physiological measurements and state anxiety levels. The mapping relationship has the potential to act as an objective measure for individualized anxiety monitoring and brain-computer interface. In detail, the prediction model proposed in the current study shows that objective physiological features measured in the resting state would help predict the state anxiety levels (Figure 5C). Therefore, for the traditional block design in the anxiety induction experiment (51, 52), as long as padding a resting state to each induction or modulation block, the state anxiety can be objectively tracked by combining the SCL and HRV features during resting state. Considering the development of portable recording devices of the physiological signals, the dynamic detection of state anxiety based on ECG and GSR can provide a more quantitative and scientific application of human anxiety-inducing models (53). Accompanied with dynamic physiological tracking, the model can enhance the efficiency and sensitivity of anxiety-related studies. By applying the prediction model on the multi-modal data during the tasks, it's possible to reconstruct the dynamic SAI changes (Supplementary Figure 1B) to quantify state anxiety with minute-level temporal resolution.

There are several limitations to this study. Introducing self-reported VAD measures achieved better prediction performance but reduced the objectiveness of the prediction model, although removing the subjective measures still allowed the model to work well. Future works that take high-dimensional central nervous system response, such as EEG, into consideration may help separate emotional response from those due to physical stimulation so as to develop a fully objectively tracking model of state anxiety levels. Moreover, pathological anxiety populations should be examined to confirm the generalization of the proposed prediction model. Besides, some emotions, such as anger, also feature high arousal and low valence. There's a chance the proposed prediction model could also predict these emotions. As the current study aimed to build a monitor tool of state anxiety with a quantitative mapping relationship between measurements and STAI-S, we only included state anxiety inventory as the dependent variable. This leads to the lack of the examination of the specificity of the model; thus, future studies are needed to test the discriminative validity of the model. Another limitation is the relatively small sample size. A more extensive population study would improve the reliability of the results to further validate the ability to use these physiological parameters as a quantitative model to evaluate anxiety levels for future studies. Future studies are needed to solve these challenges.

## Conclusion

We present a dynamic tracking model of state anxiety based on psychological and physiological data, which reflects time-resolved dynamic changes of an individual's state anxiety. The model is capable of accurately measuring state anxiety during resting state, only using objective and easy-to-acquire physiological signals.

## Data Availability Statement

The data supporting the conclusions of this article are available without undue reservation upon request to the correspondence author.

## Ethics Statement

The studies involving human participants were reviewed and approved by IRB, Shanghai Mental Health Center. The patients/participants provided their written informed consent to participate in this study.

## Author Contributions

YD, JL, XZ, and ZY contributed to the conception and design of the study, reviewed and edited the manuscript critically for an important intellectual contest. YD and JL did the experiment and data acquisition and wrote the first draft of the manuscript. YD performed the data analysis. All authors have read and approved the submitted version.

## Funding

This work was supported by National Natural Science Foundation of China (62101324, 32100885, 81971682, and 81571756), Shanghai Sailing Program (20YF1442000 and 20YF1441900), Natural Science Foundation of Shanghai (20ZR1472800), Science and Technology Commission of Shanghai Municipality (18JC1420305), Shanghai Municipal Health Commission (2019ZB0201 and 2018BR17), Gaofeng Clinical Medicine Grant Support of Shanghai Municipal Commission of Education (20171929), Shanghai Key Laboratory of Psychotic Disorders (13dz2260500), Shanghai Clinical Research Center for Mental Health (19MC1911100), Qihang Foundation of Shanghai Mental Health Center (2020-QH-01 and 2019-QH-02), the Institution-level Project of Shanghai Mental Health Center (2020-YJ01, 2020-YJ03, and 2018-YJ-02), and the Clinical Research Project of Shanghai Mental Health Center (CRC2018DSJ01-5 and CRC2019ZD04). The authors declare that this study also received funding from the Tianqiao and Chrissy Chen Institute, who was not involved in the study design, collection, analysis, interpretation of data, the writing of this article, or the decision to submit it for publication.

## Conflict of Interest

The authors declare that the research was conducted in the absence of any commercial or financial relationships that could be construed as a potential conflict of interest.

## Publisher's Note

All claims expressed in this article are solely those of the authors and do not necessarily represent those of their affiliated organizations, or those of the publisher, the editors and the reviewers. Any product that may be evaluated in this article, or claim that may be made by its manufacturer, is not guaranteed or endorsed by the publisher.
