# Supplementary material for: Dynamic Tracking of State Anxiety via Multi-Modal Data and Machine Learning
Source: Front Psychiatry. 2022 Mar 2;13:757961. doi: 10.3389/fpsyt.2022.757961 (PMC8924121; doi:10.3389/fpsyt.2022.757961)
Supplement: Supplementary file 1 [file Data_Sheet_1.docx]

Supplementary Material

**1. Comparison among regression models**

**
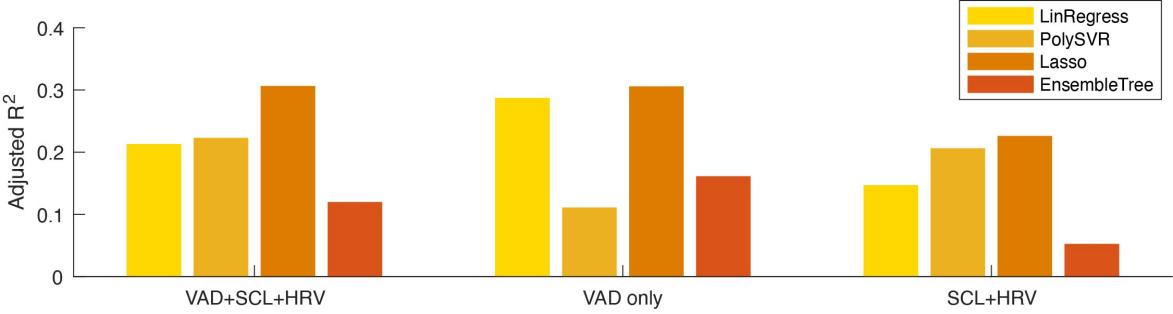
**

**Supplementary Figure 1.** Prediction performance of four regression models with all features (left), VAD only (middle), or physiological features only (right) in terms of Adjusted R^2^.

**Table 1.** Prediction performance of regression models

| **Model** | **Feature** | **Evaluation Metric** | | | |
| --- | --- | --- | --- | --- | --- |
|  |  | MAE | RMSE | R^2^ | Adjusted R^2^ |
| **Linear Regression** | VAD+SCL+HRV | 0.5966 | 0.7182 | 0.2123 | 0.2015 |
|  | VAD | 0.5677 | 0.7022 | 0.2863 | 0.2765 |
|  | SCL+HRV | 0.6445 | 0.7458 | 0.1461 | 0.1344 |
| **Support Vector Regression** | VAD+SCL+HRV | 0.6926 | 0.8426 | 0.2222 | 0.2115 |
|  | VAD | 0.6632 | 0.8378 | 0.1103 | 0.0981 |
|  | SCL+HRV | 0.6455 | 0.7617 | 0.2055 | 0.1946 |
| **LASSO Regression** | VAD+SCL+HRV | 0.5576 | 0.6789 | 0.3056 | 0.2961 |
|  | VAD | 0.5614 | 0.6892 | 0.3050 | 0.2955 |
|  | SCL+HRV | 0.6056 | 0.7104 | 0.2254 | 0.2148 |
| **Ensemble**  **of Tree** | VAD+SCL+HRV | 0.7318 | 0.9201 | 0.1191 | 0.1070 |
|  | VAD | 0.6908 | 0.8712 | 0.1606 | 0.1491 |
|  | SCL+HRV | 0.7676 | 0.9446 | 0.0517 | 0.0387 |

**2. Example application of the prediction model**


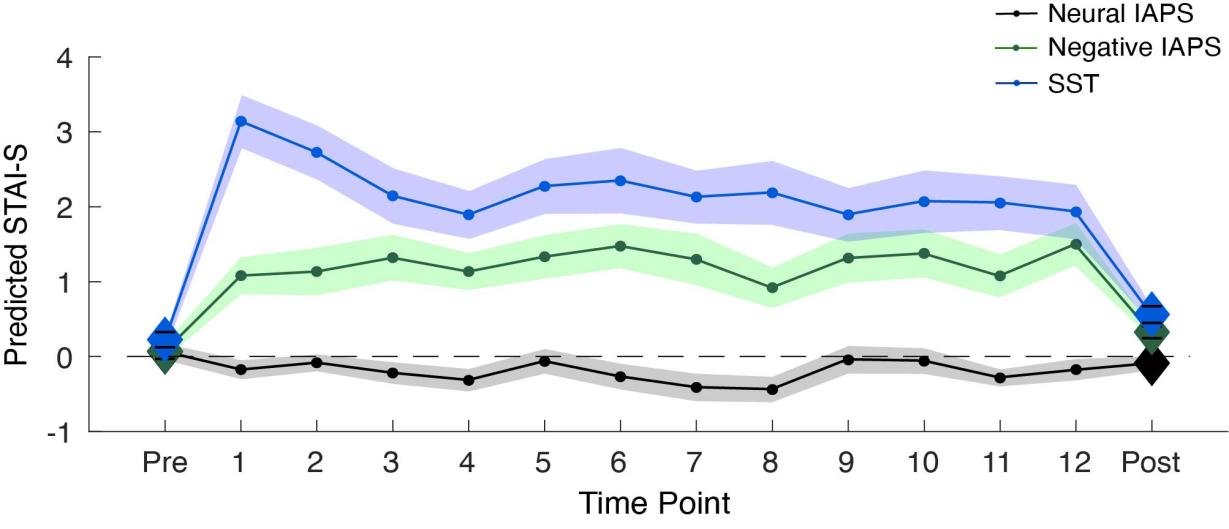


**Supplementary Figure 2.** Reconstruction of state anxiety levels during the tasks. Neutral IAPS, negative IAPS, and SST are respectively indicated by blue lines, green lines, and black lines. The dot markers correspond to each recording point; the diamond markers indicate the resting state before and after task blocks, the shadows represent the standard error mean.
